# The role of toll-like receptors in tributyltin-, dibutyltin-, and pentachlorophenol-induced stimulation of tumor necrosis factor-alpha (TNFα) production in human immune cells

**DOI:** 10.1007/s00204-026-04522-9

**Published:** 2026-08-25

**Authors:** Latoya M. Daniel, Aleshia Seaton-Terry, Ellie Bray, Meaghan Lewis, Zinia Hunter, Sophia Fisher, Brian Townsend, Margaret M. Whalen

**Affiliations:** 1 Departments of Biology, Tennessee State University, Nashville, TN 37209 USA; 2 Department of Chemistry, Tennessee State University, 3500 John A. Merritt Blvd, Nashville, TN 37209 USA

**Keywords:** Tumor Necrosis Factor, Tributyltin, Dibutyltin, Pentachlorophenol, Toll-like receptors

## Abstract

Tributyltin (TBT), Dibutyltin (DBT), and Pentachlorophenol (PCP) are persistent environmental contaminants that increase immune-cell production of pro-inflammatory cytokines and thus have the capacity to cause chronic inflammation and its associated pathologies such as cancer, cardiovascular disorders, and autoimmune conditions. TBT has been detected in human blood at concentrations up to 260 nM, DBT up to 0.3 µM, and PCP between 0.15 and 5 µM. Tumor necrosis factor-alpha (TNF-α) is a pro-inflammatory cytokine produced by immune cells in response to pathogen- or damage-associated molecular patterns (PAMPs/DAMPs) via Toll-like receptors (TLRs). Dysregulated TNF-α production contributes to chronic inflammatory pathology. Previous studies have shown that TBT, DBT, and PCP increase pro-inflammatory cytokine production by immune cells and that these increases depend on mitogen-activated protein kinases (MAPKs), which are downstream components of TLR activation. The current study indicates that TLR4 plays a role in TBT-, DBT-, and PCP-induced production of TNF-α. Additionally, TBT-induced TNF-α production was, at least in part, dependent on TLR2, DBT-induced TNF-α production on TLR1/2 and PCP-induced TNF-α production on TLR3 and TLR8. Blocking the initial component of TLR1/2, TLR2, TLR4, and TLR8 signaling, MyD88, led to very significant blockade of TBT- and PCP-induced TNF-α production. These findings provide additional understanding as to how compounds that are persistent pollutants activate production of a crucial regulator of inflammation, TNF-α, potentially contributing to chronic inflammation and its associated diseases.

## Introduction

Tumor necrosis factor alpha (TNF-α) is a key pro-inflammatory cytokine involved in managing communication among cells of innate and adaptive immune responses. TNF-α production is triggered when pathogen- or damage-associated molecular patterns (PAMPs/DAMPs) activate toll-like receptors (TLRs) on immune cells (Aggarwal et al. 2012; Kawai and Akira 2010; O’Neill and Bowie 2007; Popa et al. 2007). Inappropriately elevated levels of TNFα can lead to chronic inflammation, which is linked to several diseases, including osteoarthritis, cancer, inflammatory bowel disease, rheumatoid arthritis, Alzheimer’s disease, and cardiovascular disease (Adegbola et al. 2018; Balkwill and Mantovani 2001; Barcia et al. 2011; Farrugia and Baron 2016; Fischer and Maier 2015; Frankola et al. 2011; Macarthur et al. 2004; Rauf et al. 2022).

Tributyltin (TBT) and dibutyltin (DBT) are organotin compounds found to contaminate the environment. TBT has been used as a biocide in antifouling paints, disinfecting industrial cooling water, textiles, baking parchments, sponges, and other household items (Antizar-Ladislao 2008; Kimbrough 1976; Laughlin Jr and Lindén 1985; Roper 1992; Takahashi et al. 1999; Yamada et al. 1993), and has been found in human blood at levels as high as 260 nM (Kannan et al. 1999; Whalen et al. 1999). It increases pro-inflammatory cytokine production (interleukin (IL)-1β, IL-6, interferon γ (IFNγ), and tumor necrosis factor α (TNF α) by human immune cells (Brown et al. 2018; Lawrence et al. 2018). This TBT stimulation of pro-inflammatory cytokines is dependent on mitogen activated protein kinases (MAPKs) (Brown et al. 2018; Lawrence et al. 2018), which are components of TLR signaling pathways. Recently, it was shown that TLR1/2, TLR4, and TLR 8 were needed for TBT to cause increases in the pro-inflammatory cytokines IL-1β and IL-6 (Alcala et al. 2022). DBT is used primarily as a catalyst and stabilizer in the production of polyvinyl chloride (PVC) products (Chantong et al. 2014; Wu et al. 2023), but also as a deworming agent in poultry (Epstein et al. 1991) with levels as high as 0.3 µM detected in human blood (Kannan et al. 1999; Whalen et al. 1999). DBT, like TBT, causes MAPK-dependent stimulation of IL-1β and IL-6 (Brown et al. 2018; Lawrence et al. 2018; Sushak et al. 2020). Furthermore, the TLR adaptor MyD88 was needed for DBT to stimulate increases in these cytokines (Seaton-Terry et al. 2025).

Pentachlorophenol (PCP) is an organochlorine used as a wood preservative, fungicide, and pesticide (Cirelli 1978; Ingerman et al. 2001) and is present in human serum at 0.26 to 5 µM in individuals with known exposure, while those without obvious exposure displayed average levels of 0.15 µM (Cline et al. 1989). It is associated with increased risk of non-Hodgkin lymphoma and kidney cancer (Cooper and Jones 2008; Demers et al. 2006; Guyton et al. 2016). PCP increases the production of pro-inflammatory cytokines, IL-1β and IL-6 (Martin et al. 2019a, b; Martin et al. 2019a, b) and this PCP-induced stimulation of IL-1β and IL-6 production, like that seen with TBT and DBT, is dependent upon MAPKs (Martin et al. 2019a, b; Martin et al. 2019a, b) and requires TLRs and the TLR adapter, MyD88 (Seaton-Terry et al. 2025).

Based on the importance of TLR pathways in TBT-, DBT-, and PCP-induced stimulation of IL-1β and IL-6, the current study investigates their role in stimulation of TNFα seen with exposures to these same compounds. Due to their persistence in the environment and potential health risks, it is important to understand how these compounds can lead to aberrant elevation of inflammatory cytokines. As mentioned earlier, inappropriate elevation of a potent pro-inflammatory cytokine such as TNFα can result in chronic inflammation, which is associated with many disease states (Adegbola et al. 2018; Balkwill and Mantovani 2001; Barcia et al. 2011; Farrugia and Baron 2016; Fischer and Maier 2015; Frankola et al. 2011; Macarthur et al. 2004; Rauf et al. 2022).

## Materials and methods

### Cell preparation

Peripheral blood mononuclear cells (PBMCs) were isolated from leukocyte filters obtained from the Red Cross Blood Bank Facility in Nashville, TN (Meyer et al. 2005). The filters were back-flushed with sterile phosphate-buffered saline (PBS) containing 5 mM disodium EDTA and 2.5% [w/v] sucrose, and the resulting eluent was collected. The eluent was layered onto Lymphosep™ - Lymphocyte Separation Medium (1.077 g/mL) (Fisher Scientific, Pittsburg, PA) and centrifuged at 1200 g for 30 min. During centrifugation, granulocytes and erythrocytes pelleted at the bottom of the tube. PBMCs (lymphocytes and monocytes) form a layer at the interface between the eluting medium and Lymphosep™. The PBMC layer was collected and washed with phosphate buffered saline, pH 7.4 (PBS) (500 g, 10 min). To remove platelets, the washed cells were layered onto bovine calf serum and the PBMCs were pelleted by centrifugation. The PBMC pellet was resuspended in RPMI-1640 complete medium, which contained RPMI-1640 supplemented with 10% heat-inactivated bovine calf serum (BCS), 2 mM L-glutamine, 50 U/mL penicillin G, and 50 µg/mL streptomycin.

### Chemical preparation

Tributyltin chloride (TBT), dibutyltin dichloride (DBT), and pentachlorophenol (PCP) were obtained from Sigma-Aldrich (96%, St. Louis, MO) and Thermo Fisher Scientific (Waltham, MA). TBT, supplied as a neat standard, was dissolved in deionized water to create a 1 mM stock solution, which was then diluted into cell culture media to achieve the desired concentrations. DBT stock solution was prepared by dissolving DBT in dimethyl sulfoxide (DMSO) to a final concentration of 0.685 M, followed by appropriate dilution into cell culture media and including and appropriate DMSO control. PCP stock solution was prepared at a concentration of 100 mM in DMSO, with desired concentrations obtained by diluting the stock into cell culture media.

Inhibitors were obtained from Fisher Scientific. TLR2, TLR4, and TLR8 inhibitors (C29, TAK242, and CUCPT9A) were initially prepared as 50 mM stock solutions in DMSO and then diluted to a final concentration of 10 µM. The stock solutions were diluted in cell culture media to give final concentrations of 10 µM for the C29, TAK242, and CUCPT9a. Concentrations were based on previously published studies (Bhattacharyya et al. 2018; Mistry et al. 2015; Zhang et al. 2018). Similarly, inhibitors targeting the TLR3 and TLR 1/2 pathways (CUCPT4A and CUCPT22) were prepared as 50 mM stock solutions in DMSO and further diluted to achieve final concentrations of 20 µM for CUCPT4A and 8 µM for CUCPT22. These concentrations were determined based on previously published research (Cheng et al. 2012; Faksh et al. 2016). The MyD88 inhibitor, TJ-M2010-5 was diluted to give a final concentration of 10 µM (concentration based on those used in previous studies (Miao et al. 2020). Appropriate DMSO controls were prepared for each inhibitor.

### Cell treatment and lysis

Peripheral blood mononuclear cells (PBMCs) were pretreated with a specific inhibitor or the corresponding control for 1 h before exposure to tributyltin (TBT) at concentrations of 25 nM to 100 nM, pentachlorophenol (PCP) at 1 µM to 5 µM, or dibutyltin (DBT) at 0.1 µM to 0.5 µM, along with an appropriate control, for 24 h. Following treatment, the cells were centrifuged to collect the supernatant, and each resulting cell pellet was lysed using 150 µL of lysis buffer (M-PER™ mammalian protein extraction reagent from Thermo Scientific, Rockford, IL) per 4 million cells. The supernatants and cell lysates were immediately stored at − 80 °C until further analysis by ELISA or Western blotting.

### TNF-α secretion assay

The levels of secreted TNF-α were quantified using OptEIA™ enzyme-linked immunosorbent assay (ELISA) kit (BD-Pharmingen, San Diego, CA). Ninety-six well flat-bottomed plates (ELISA-optimized, Fisher, Pittsburgh, PA) were coated with the TNF-α capture antibody and incubated overnight at 4 °C. After incubation, the plate was washed, and non-specific binding was blocked using bovine calf serum (BCS) diluted 1:10 in PBS. Supernatants from treated and control cells, appropriately diluted, along with TNF-α standards, were then added to the wells and incubated for 2 h. Following this, the detection antibody was added and incubated for 1 h, after which the substrate was introduced. Substrates reacted to produce a color change, and absorbance was measured at 450 nm using a Thermo Labsystems Multiskan MCC/340 plate reader (Fisher Scientific, Pittsburgh, PA).

### Western blot

To analyze cellular lysates, samples were subjected to 10% sodium dodecyl sulfate-polyacrylamide gel electrophoresis (SDS-PAGE). The separated proteins were then transferred onto a PVDF membrane and immunoblotted with antibodies against TNF-α and β-actin (loading control) (Cell Signaling Technologies, Beverly, MA). Antibody binding was detected using a UVP Image Station (UVP, LLC, Upland, CA). Protein expression differences were assessed by comparing compound-treated samples to their appropriate control. β-actin expression was evaluated for each treatment to ensure uniform protein loading. The intensity of each protein band was normalized to β-actin to account for minor variations in protein loading across lanes.

### Statistical analysis

Statistical significance was determined using Student’s t-test, with type I error controlled by the Bonferroni-Holm method (Holm 1979). In this approach, p-values were ranked from lowest to highest and sequentially compared to α/m (where m represents the total number of comparisons), followed by α/(m-1), …, α/2, α/1.

## Results

### Effects of inhibiting TLR1/2, TLR2, and TLR4 on TBT-induced TNF-α production

**Fig. 1:**
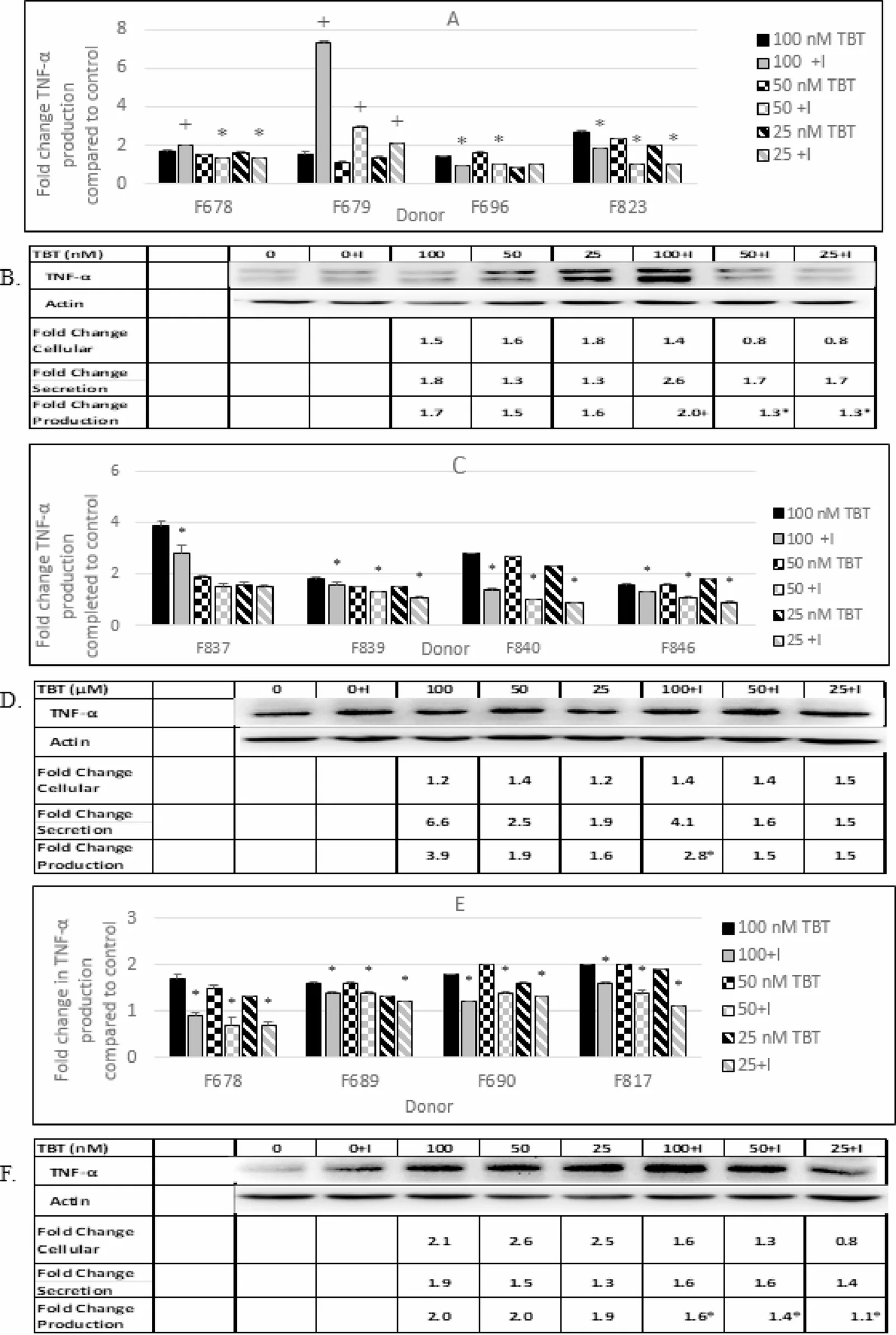
Effects of TLR1/2, TLR2, and TLR4 inhibition on TBT-induced production of TNF-α. PBMCs were treated with TLR1/2-, TLR2-, or TLR4-selective inhibitors or appropriate controls for 1 h prior to exposure to 100, 50, 25 and 0 (control) nM TBT. **A** TLR1/2 inhibitor (I=CUCPT22, 8 µM): Changes in fold production for cells from 4 distinct donors. Fold changes in intracellular levels were determined by western blot and fold changes for secreted levels were determined by ELISA for each cell treatment for each donor. An increase in secretion or in intracellular levels is a number greater than 1; a decrease is a number less than 1. The control is arbitrarily set at 1. Average of the intracellular and secreted fold changes greater than 1indicates an increase in production. **B** TLR1/2 inhibitor: Blot from a representative experiment (F678) showing fold increases in intracellular levels, secreted levels, and production of TNF-α. **C** TLR2 inhibitor (I=C29, 10 µM): Changes in fold production for cells from 4 distinct donors. **D** TLR2 inhibitor: Blot from a representative experiment (F837). **E** TLR4 inhibitor (I=TAK242, 10 µM): Changes in fold production for cells from 4 distinct donors. **F)** TLR4 inhibitor: Blot from a representative experiment (F817). An asterisk (*) indicates a significant decrease and a plus sign (+) indicates a significant increase in fold production in the presence of the TLR inhibitor (*P* < 0.0167 initially, using the Bonferroni-Holm method)

TLR1/2, TLR2, and TLR4 are predominantly cell surface receptors on immune cells. PBMCs isolated from 4 distinct donors were pre-treated with a selective inhibitor or an appropriate control for 1 h and then exposed to TBT at 100, 50, and 25 nM for 24 h. Inhibition of TLR1/2 did not consistently diminish the TBT-induced increases in TNF-α production (Fig. 1A and B). For example, cells from F678 showed 1.7-, 1.5-, and 1.6-fold increases in TNF-α production when exposed to 100, 50, and 25 nM TBT in the absence of the TLR1/2 inhibitor. In the presence of the inhibitor, 100 nM TBT showed enhanced ability to increase TNF-α production, while exposures at 50 and 25 nM showed decreased ability to stimulate TNF-α production (Fig. 1B). Results from cells from 3 additional donors indicate that the role of TLR1/2 in TBT-induced increases in TNF-α production varies within and among donors (Fig. 1A).

TBT-induced increases in TNF-α production were diminished when TLR2 was inhibited (Fig. 1C and D.) For example, cells from donor F840 exhibited 2.8-, 2.7-, and 2.3- fold increases in TNF-α production in response to TBT exposures (100, 50, and 25 nM) in the absence of TLR2 inhibitor. When TLR2 was inhibited, the TBT-stimulated increase at 100 nM was reduced to 1.4-fold and there were no increases at 50 or 25 nM. Similar results were seen with cells from the other donors. These findings indicate that TLR2 is, at least in part, involved in TBT-induced stimulation of TNF-α production by human immune cells.

Inhibition of TLR4 (using TAK242) consistently diminished TBT-induced increases in TNF-α production in cells from all donors (Fig. 1E and F). For example, cells from F678 showed 2.0-, 2.0-, and 1.9-fold increases when exposed to 100, 50, 25 nM TBT in the absence of TLR4 inhibitor. When TLR4 inhibitor was present, there was diminished TNF-α production at those same TBT exposures (1.6, 1.4, and 1.1-fold, respectively) (Fig. 1F). These findings indicate a role for TLR4 in the TBT-induced production of TNF-α by human immune cells.

### Effects of inhibiting TLR3 and TLR8 on TBT-induced TNF-α production

**Fig. 2:**
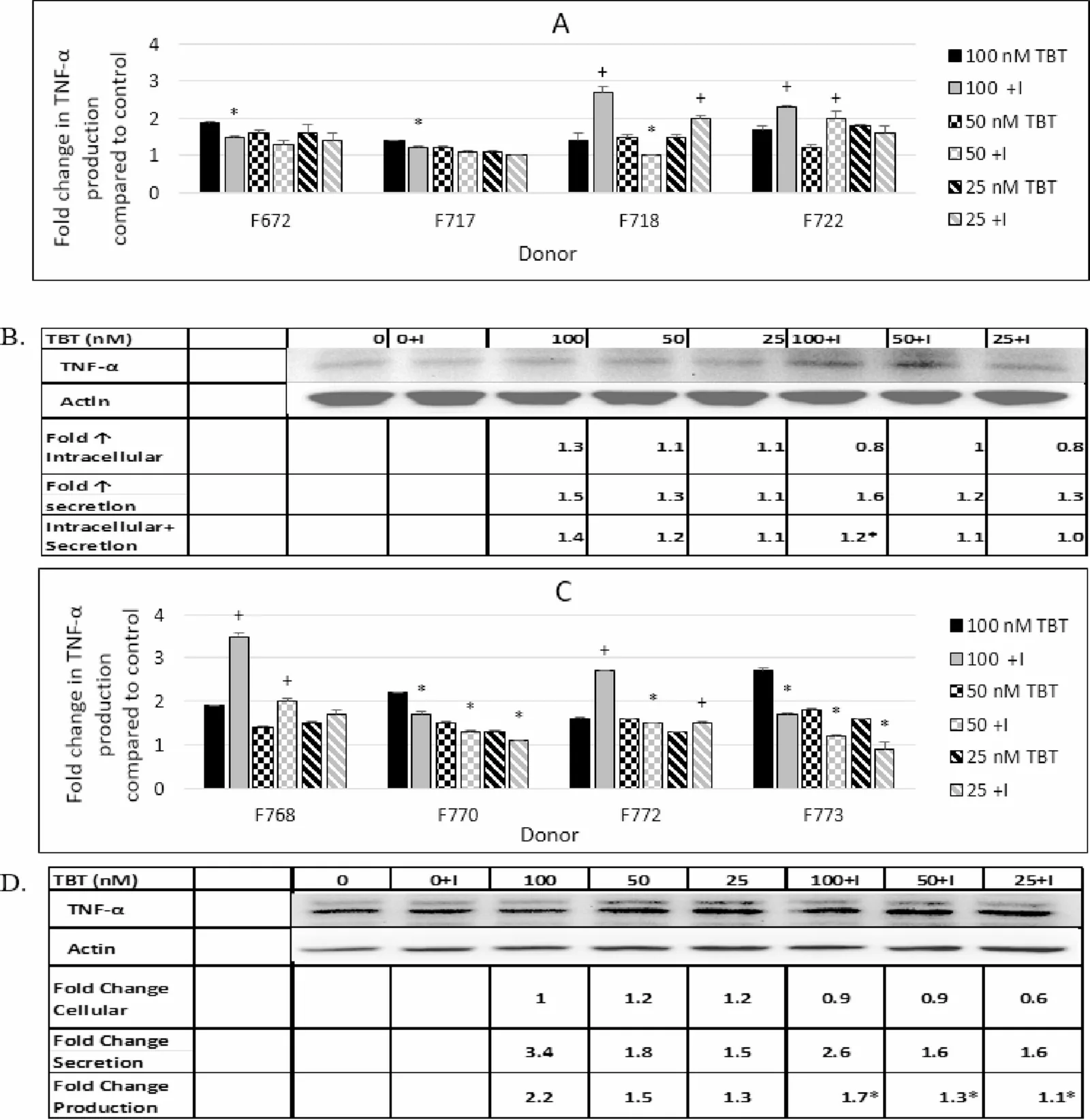
Effects of TLR3 and TLR8 inhibition on TBT-induced production of TNF-α. PBMCs were treated with a TLR3- or TLR8-selective inhibitor or appropriate control for 1 h prior to exposure to 100, 50, 25 and 0 (control) nM TBT. **A** TLR3 inhibitor (I=CUCPT4a, 20 µM): Changes in fold production for cells from 4 distinct donors. **B** TLR3 inhibitor: Blot from a representative experiment (F717) showing fold increases in intracellular levels, secreted levels, and production of TNF-α. **C** TLR8 inhibitor (I=CUCPT9a, 10 µM): Changes in fold production for cells from 4 distinct donors. **D** TLR8 inhibitor: Blot from a representative experiment (F770). An asterisk (*) indicates a significant decrease and a plus sign (+) indicates a significant increase in fold production in the presence of the TLR inhibitor (*P* < 0.0167 initially, using the Bonferroni-Holm method)

In contrast to TLR1/2, 2, and 4, TLRs 3 and 8 are intracellular receptors located in the endosome. Inhibition of TLR3 did not consistently diminish TBT-induced increases in TNF-α production (Fig. 2A and B). In the absence of the TLR3 inhibitor, cells from donor F717 demonstrated 1.4-, 1.2-, and 1.1-fold increases in TNF-α production when exposed to 100, 50, and 25 nM TBT, respectively. In the presence of the TLR3 inhibitor, a significant reduction in TBT-induced TNF-α production was observed at 100 nM TBT (Fig. 2B). In contrast, cells from donor F718 demonstrated significant increases in TBT-induced TNF-α production in the presence of TLR3 inhibitor.

As was seen with inhibition of TLR3, the effects of selective inhibition of TLR8 (CUCPT9a) varied across donors with some donors showing decreased TBT-induced elevations while others showed increased effectiveness of TBT (Fig. 2C and D). Thus, TLR 8 is not consistently involved in TBT stimulation of TNF-α production by immune cells.

### Effects of inhibiting TLR1/2, TLR2, and TLR4 on DBT-induced TNF-α production

**Fig. 3:**
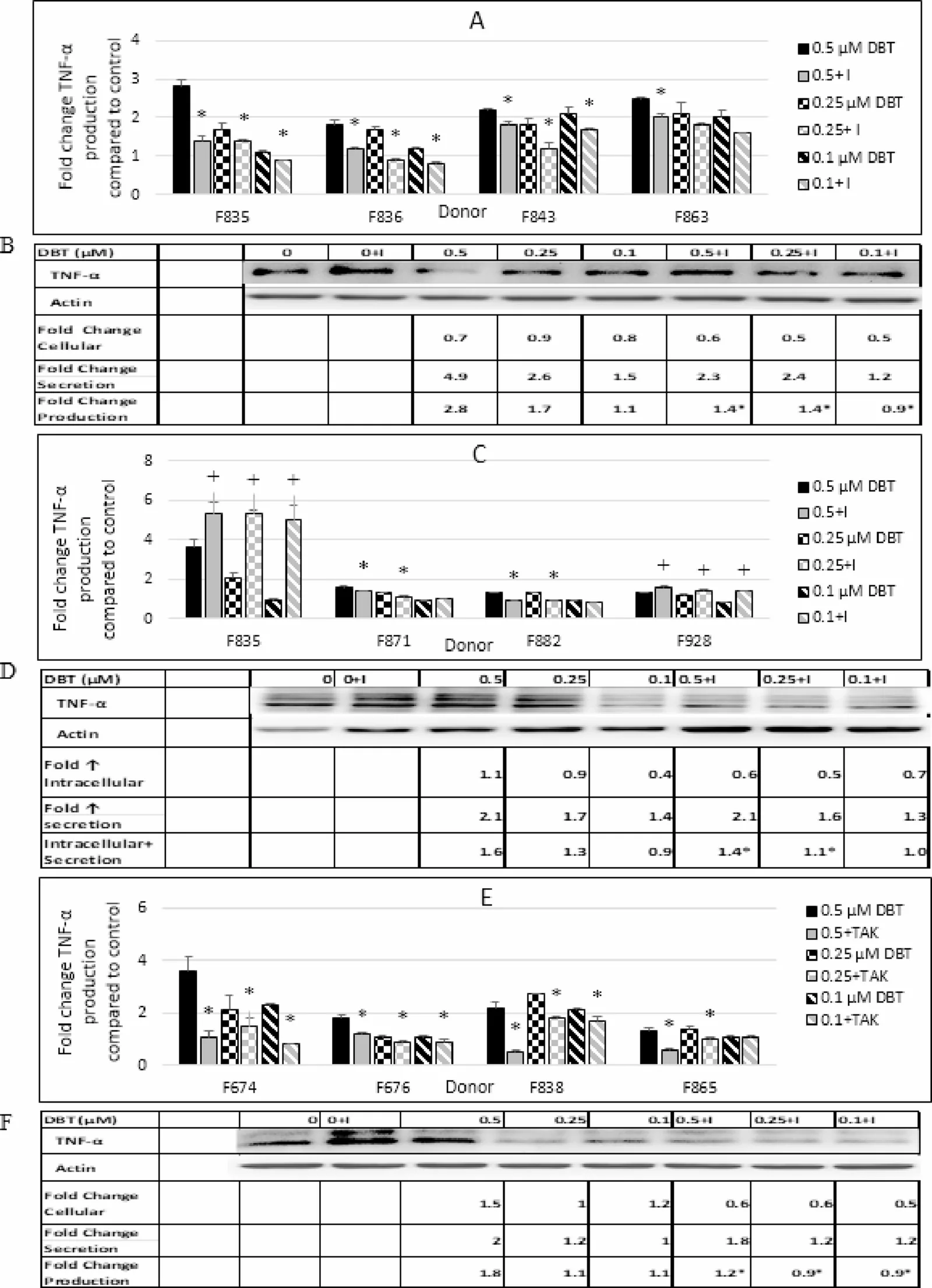
Effects of TLR1/2, TLR2, and TLR4 inhibition on DBT-induced production of TNF-α. PBMCs were treated with a TLR1/2-, TLR2-, or TLR4-selective inhibitor or appropriate control for 1 h prior to exposure to 0.5, 0.25, 0.1 and 0 (control) µM DBT. **A** TLR1/2 inhibitor (I=CUCPT22, 8 µM): Changes in fold production for cells from 4 distinct donors (as described in Fig. 1). **B** TLR1/2 inhibitor: Blot from a representative experiment (F835) showing fold increases in intracellular levels, secreted levels, and production of TNF-α. **C** TLR2 inhibitor (I=C29, 10 µM): Changes in fold production for cells from 4 distinct donors. **D** TLR2 inhibitor: Blot from a representative experiment (F871). **E** TLR4 inhibitor (I=TAK242, 10 µM): Changes in fold production for cells from 4 distinct donors. **F** TLR4 inhibitor: Blot from a representative experiment (F676). An asterisk (*) indicates a significant decrease and a plus sign (+) indicates a significant increase in fold production in the presence of the TLR inhibitor (*P* < 0.0167 initially, using the Bonferroni-Holm method)

The effects of selectively inhibiting TLR1/2 on DBT-stimulated TNF-α production are shown in Fig. 3A and B. Cells were incubated with inhibitor or an appropriate control for 1 h and then exposed to DBT at 0.5, 0.25, and 0.1 µM for 24 h. The presence of the TLR1/2 inhibitor consistently diminished the DBT-induced increases in TNF-α levels at one or more DBT concentration in cells from all donors (Fig. 3A). For example, cells from donor F835 showed 2.8-, 1.7-, and 1.1-fold increases in TNF-α production when exposed to 0.5, 0.25, and 0.1 µM DBT, in the absence of TLR1/2 inhibitor. In the presence of the TLR1/2 inhibitor, the same DBT exposures resulted in diminished DBT stimulation of TNF-α production (1.4-, 1.4- fold, and no increase) (Fig. 3B). These findings indicate that TLR1/2 plays a part in mediating DBT-induced increases in TNF-α production by human immune cells.

Inhibition of TLR 2 did not consistently diminish DBT-induced increases in TNF-α production (Fig. 3C and D). For example, cells from donor F871 showed 1.6- and 1.3-fold increases in TNF-α production at 0.5 and 0.25 µM DBT, which were decreased in the presence of the inhibitor (Fig. 3D). In contrast, cells from donor F835 showed increased ability of DBT to stimulate TNF-α production in the presence of TLR2 inhibitor (Fig. 3C).

TLR4 inhibition consistently diminished the DBT-induced increases in TNF-α levels in cells from all donors (Fig. 3E and F). For example, cells from donor F676 showed 1.8-, 1.1-, and 1.1-fold increase in TNF-α production when exposed 0.5, 0.25, and 0.1 µM DBT, in the absence of TLR4 inhibitor. In the presence of the TLR4 inhibitor, the same DBT exposures showed diminished DBT stimulation of TNF-α production (1.2-fold at 0.5 µM and no increase at 0.25 and 0.1 µM) (Fig. 3F). These findings indicate that TLR4 has a role in mediating DBT-induced increases in TNF-α production by human immune cells.

### Effects of inhibiting TLR3 and TLR8 on DBT-induced TNF-α production

**Fig. 4:**
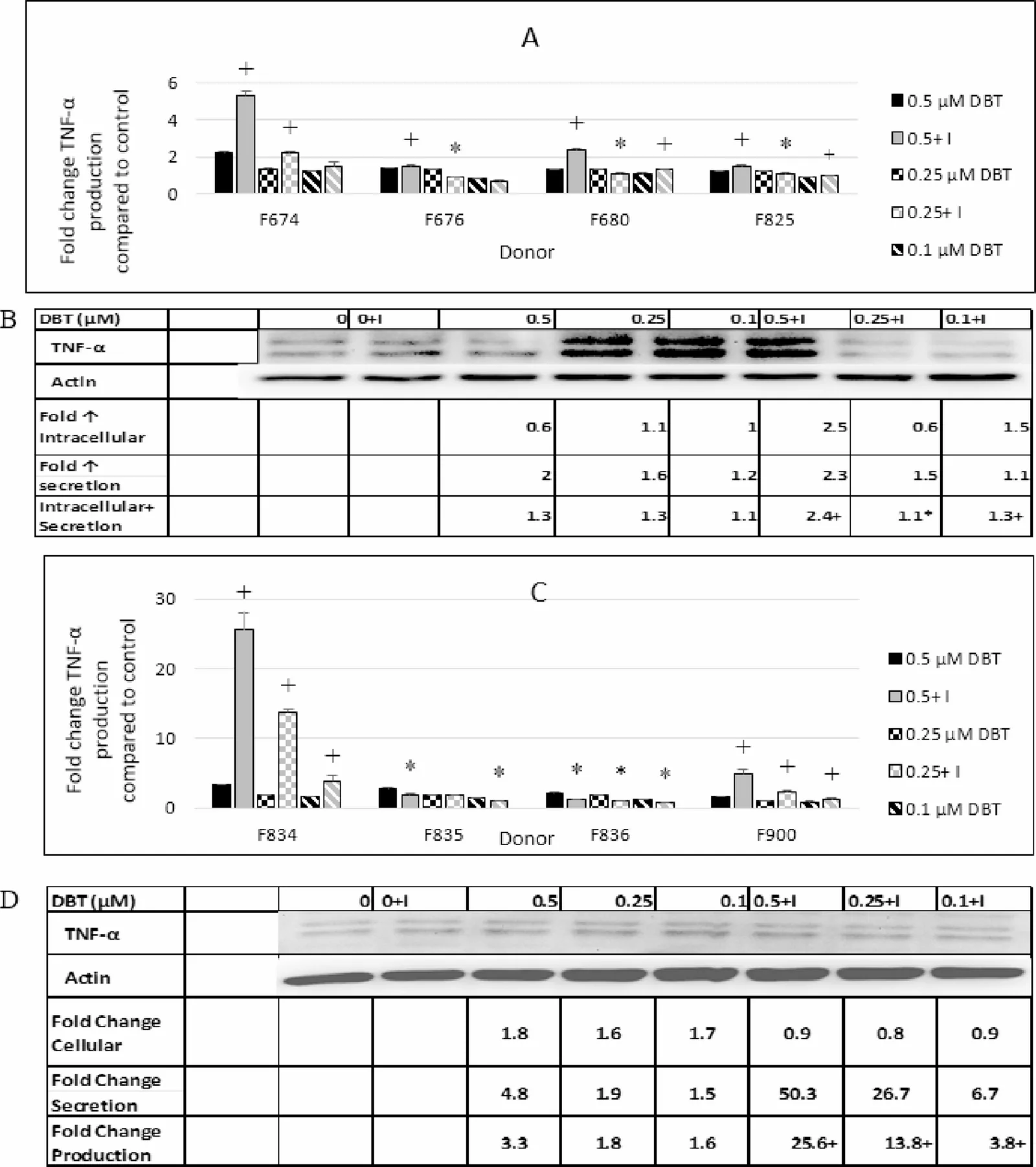
Effects of TLR3 and TLR8 inhibition on DBT-induced production of TNF-α. PBMCs were treated with a TLR3- or TLR8-selective inhibitor or appropriate control for 1 h prior to exposure to 0.5, 0.25, 0.1 and 0 (control) µM DBT. **A** TLR3 inhibitor (I=CUCPT4a, 20 µM): Changes in fold production for cells from 4 distinct donors. **B** TLR3 inhibitor: Blot from a representative experiment (F674) showing fold increases in intracellular levels, secreted levels, and production of TNF-α. **C** TLR8 inhibitor (I=CUCPT9a, 10 µM): Changes in fold production for cells from 4 distinct donors. **D** TLR8 inhibitor: Blot from a representative experiment (F834). An asterisk (*) indicates a significant decrease and a plus sign (+) indicates a significant increase in fold production in the presence of the TLR inhibitor (*P* < 0.0167 initially, using the Bonferroni-Holm method)

Selective inhibition of TLR3 with CUCPT4a did not consistently diminish DBT-induced increases in TNF-α production (Fig. 4A and B). For example, cells from donor F680 showed fold changes of 1.3, 1.3, and 1.1 in TNF-α production when exposed to DBT (Fig. 4B). In the presence of the TLR3 inhibitor, TNF-α increased to 2.4 and 1.3-fold when exposed to 0.5 and 0.1 µM DBT. Cells from all donors exhibited increases in TNF-α production in the presence of TLR3 inhibitor at one or more DBT exposure.

As was seen with TLR3, inhibition of TLR8 did not consistently diminish DBT-induced increases in TNF-α production (Fig. 4C). For example, cells from donor F834 exhibited 3.3-, 1.8-, and 1.6-fold increases in TNF-α production when exposed to 0.5, 0.25, 0.1 µM DBT, respectively. In the presence of the inhibitor, these DBT-induced increases in TNF-α production were enhanced (Fig. 4D).

### Effects of inhibiting TLR1/2, TLR2, and TLR4 on PCP-induced TNF-α production

**Fig. 5:**
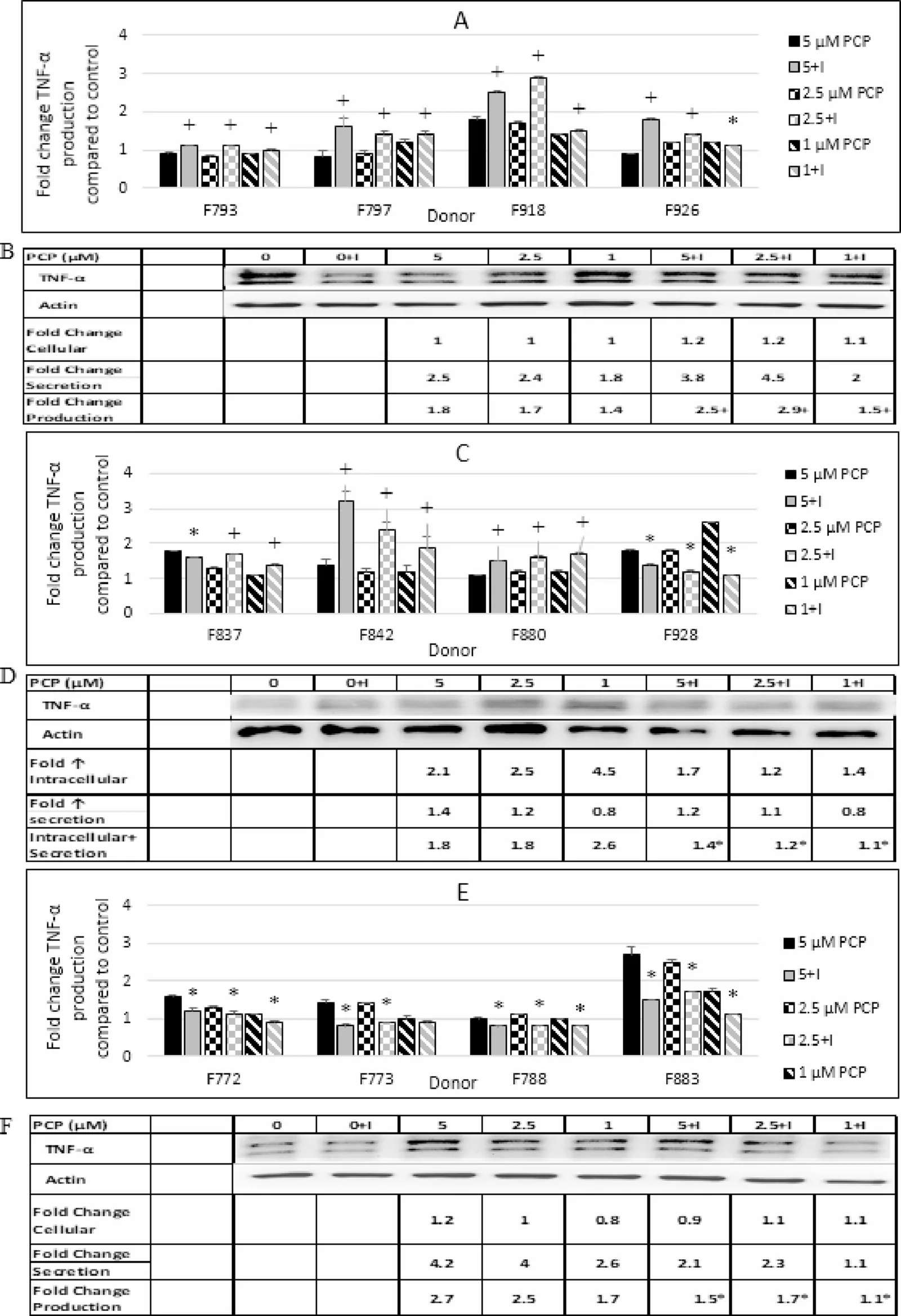
Effects of TLR1/2, TLR2, and TLR4 inhibition on PCP-induced production of TNF-α. PBMCs were treated with a TLR1/2-, TLR2-, or TLR4-selective inhibitor or appropriate control for 1 h prior to exposure to 5, 2.5, 1 and 0 (control) µM PCP. **A** TLR1/2 inhibitor (I=CUCPT22, 8 µM): Changes in fold production for cells from 4 distinct donors (as described in Fig. 1). **B** TLR1/2 inhibitor: Blot from a representative experiment (F918) showing fold increases in intracellular levels, secreted levels, and production of TNF-α. **C** TLR2 inhibitor (I=C29, 10 µM): Changes in fold production for cells from 4 distinct donors. **D** TLR2 inhibitor: Blot from a representative experiment (F928). **E** TLR4 inhibitor (I=TAK242, 10 µM): Changes in fold production for cells from 4 distinct donors.** F** TLR4 inhibitor: Blot from a representative experiment (F883). An asterisk (*) indicates a significant decrease and a plus sign (+) indicates a significant increase in fold production in the presence of the TLR inhibitor (*P* < 0.0167 initially, using the Bonferroni-Holm method)

Cells from 4 distinct donors were incubated with TLR1/2 selective inhibitor (CUCPT22) or an appropriate control for 1 h and then exposed to PCP at 5, 2.5, and 1 µM for 24 h. As was seen with TBT, inhibition of TLR1/2 did not consistently diminish the PCP-induced increases in TNF-α production (Fig. 5A and B). For example, cells from donor F918 showed 1.8-, 1.7-, and 1.4-fold increases in TNF-α when exposed to 5, 2.5, and 1 µM PCP. In the presence of the inhibitor, all PCP exposures resulted in enhanced PCP stimulation of TNF-α production (Fig. 5B).

When TLR 2 was inhibited, PCP-induced increases in TNF-α production were not consistently diminished (Fig. 5C and D). For example, cells from donor F928 showed a 1.8-, 1.8-, and 2.6-fold increase in TNF-α production at 5, 2.5, and 1 µM PCP. In the presence of the inhibitor, there were significant decreases in PCP-induced TNF-α production (1.4-, 1.2-, 1.1-fold) (Fig. 5D). However, the cells from donors F837 and F842 exhibited an enhanced ability to increase TNF-α production at all concentrations of PCP (5, 2.5, and 1 µM) in the presence of the TLR 2 inhibitor (Fig. 5D).

PCP stimulation of TNF-α production was decreased in cells from all donors when TLR4 was inhibited with TAK242 (Fig. 5E and F). For example, cells from F883 showed 2.7-, 2.5-, and 1.7-fold increases when exposed to 5, 2.5, 1 µM PCP without inhibitor. When inhibitor was present, these same PCP exposures were far less effective at increasing TNF-α production (increases of 1.5, 1.7, and 1.1fold, respectively) (Fig. 5F). Similar results were observed in cells from all donors (Fig. 5E). Thus, TLR4 plays a role in PCP-induced stimulation of TNF-α production.

### Effects of inhibiting TLR3 and TLR8 on PCP-induced TNF-α production

**Fig. 6:**
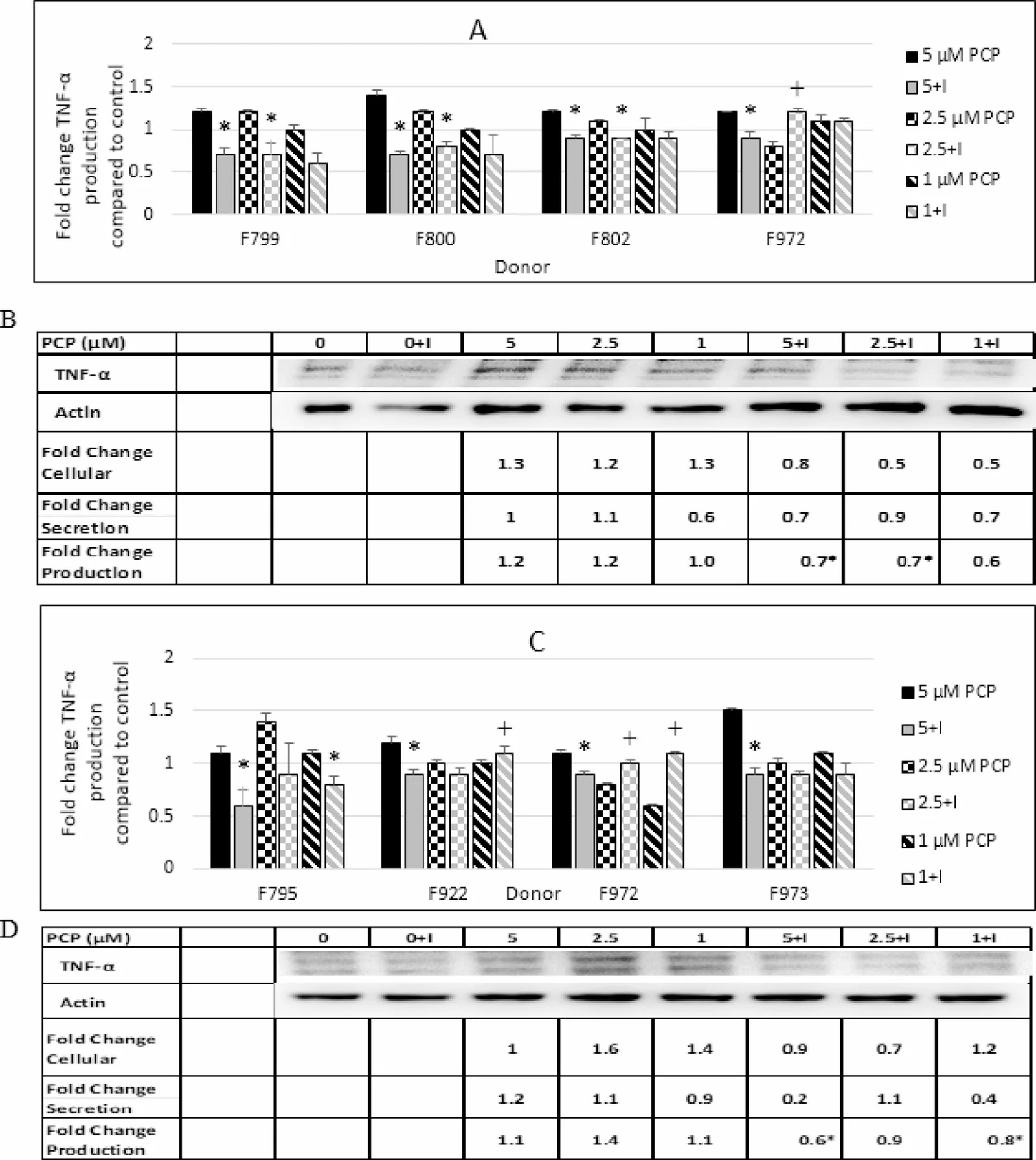
Effects of TLR3 and TLR8 inhibition on PCP-induced production of TNF-α. PBMCs were treated with a TLR3- or TLR8-selective inhibitor or appropriate control for 1 h prior to exposing the cells to 5, 2.5, 1 and 0 (control) µM PCP. **A** TLR3 inhibitor (I=CUCPT4a, 20 µM): Changes in fold production for cells from 4 distinct donors. **B** TLR3 inhibitor: Blot from a representative experiment (F799) showing fold increases in intracellular levels, secreted levels, and production of TNF-α. **C** TLR8 inhibitor (I=CUCPT9a, 10 µM): Changes in fold production for cells from 4 distinct donors. **D** TLR8 inhibitor: Blot from a representative experiment (F795). An asterisk (*) indicates a significant decrease and a plus sign (+) indicates a significant increase in fold production in the presence of the TLR inhibitor (*P* < 0.0167 initially, using the Bonferroni-Holm method)

In the presence of TLR3 inhibitor, PCP-induced increases in TNF-α production were decreased in cells from all donors (Fig. 6A and B). For example, cells F799 exhibited increases in TNF-α production of 1.2 and 1.2, when exposed to 5 and 2.5 µM PCP in the absence of inhibitor, while in the presence of inhibitor no PCP-induced increases in TNF-α production were seen (Fig. 6B). These data indicate that TLR 3 plays a role in PCP stimulation of TNF-α production.

Inhibition of TLR8 diminished PCP-induced increases in TNF-α production in cells from all donors at the 5 µM exposure (Fig. 6D and E). These findings suggest that TLR8 is needed for PCP-induced TNFα production, but only at the highest exposure level.

### Effects of MyD88 inhibition on TBT-, DBT-, and PCP-induced TNF-α production

**Fig. 7:**
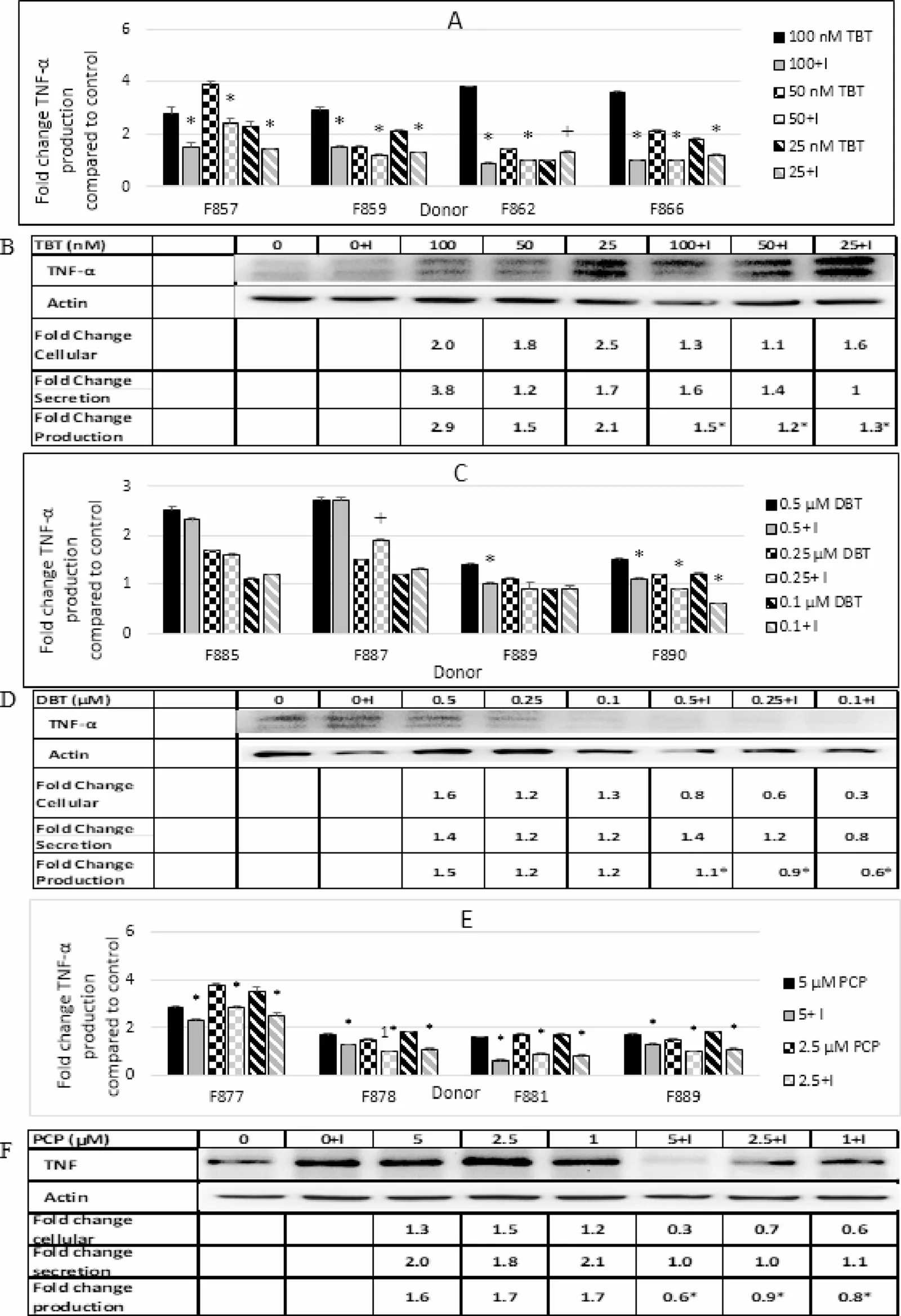
Effects of MyD88 inhibition on TBT-, DBT-, and PCP-induced production of TNF-α. PBMCs were treated with 10 µM a selective MyD88 inhibitor (I = TJ-M2010-5) or appropriate control for 1 h prior to exposure to either TBT (100, 50, 25 nM), DBT (0.5, 0.25, 0.1µM) or PCP (5, 2.5, 1 µM) for 24 h. **A** TBT-induced production of TNF-α: Changes in fold production for cells from 4 distinct donors. **B** TBT-induced production of TNF-α: Blot from a representative experiment (donor F859) showing fold increases in intracellular levels, secreted levels, and production of TNF-α. **C** DBT-induced production of TNF-α: Changes in fold production for cells from 4 distinct donors. **D** DBT-induced production of TNF-α: Blot from a representative experiment (F890) showing fold increases in intracellular levels, secreted levels, and production of TNF-α. **E** PCP-induced production of TNF-α: Changes in fold production for cells from 4 distinct donors. **F** PCP-induced production of TNF-α: Blot from a representative experiment (F881) showing fold increases in intracellular levels, secreted levels, and production of TNF-α. An asterisk (*) indicates a significant decrease and a plus sign (+) indicates a significant increase in fold production in the presence of the MyD88 inhibitor (*P* < 0.0167 initially, using the Bonferroni-Holm method)

The effects of inhibiting MyD88 on TBT-stimulation TNF-α production in PBMCs from four donors are shown in Fig. 7A and B. PBMCs were treated with a selective MyD88 inhibitor (TJ-M2010-5) or an appropriate DMSO control for 1 h prior to exposing the cells to 100, 50, 25 and 0 (control) nM TBT. When MyD88 was blocked, the ability of TBT to cause increased production of TNF-α was very significantly diminished. This indicates MyD88 dependence. These results verify the involvement of the 2 cell-surface TLRs, TLR2 and TLR4 in TBT-induced stimulation of TNF-α production, as both of these receptors are linked to the MyD88 adapter.

In contrast to the results with TBT, when MyD88 was blocked the ability of DBT to cause increased production of TNF-α was diminished at one or more DBT exposure in 2 of the 4 donors tested but not in the cells from the other 2 donors (Fig. 7C and D). Thus, while TLR1/2 and TLR4 have a role in DBT-induced elevations of TNF-α, this is not the only route by which these increases occur.

As was seen with TBT, when MyD88 was blocked, PCP’s ability to elevate TNF-α production was either greatly impeded or completely blocked Fig. 7E and F. This is consistent with the role of TLR4 in PCP-induced TNF-α production by PBMCS described earlier. Thus, PCP functions as a MyD88-TLR4 activator, inducing TNF-α.

## Discussion

TLRs regulate the production of pro-inflammatory cytokines, such as TNF-α, in response to injury or infection by binding DAMPs and PAMPs (Gaestel et al. 2009; Medzhitov 2001). Activated TLRs recruit adaptor proteins, MyD88 and TRIF, which direct the intracellular signaling response leading to pro-inflammatory cytokine production. TNF-α is produced by macrophages, lymphocytes, and other immune cells in response to TLR activation (Locksley et al. 2001) and plays a central role in coordinating inflammatory responses by promoting leukocyte recruitment, fever induction, and acute-phase protein synthesis (Van Loo and Bertrand 2023; Zelová and Hošek 2013). Under normal physiological conditions, TNF-α expression is transient and tightly regulated (Varfolomeev and Vucic 2018). Chronic elevation of TNF-α disrupts immune balance, promoting persistent inflammation associated with autoimmune disorders, neurodegenerative disease, and carcinogenesis (Adegbola et al. 2018; Balkwill and Mantovani 2001; Barcia et al. 2011; Farrugia and Baron 2016; Fischer and Maier 2015; Frankola et al. 2011; Macarthur et al. 2004; Rauf et al. 2022). Exposure to TBT increases TNF-α production by human immune cells and these increases are dependent on MAPKs (Lawrence et al. 2018), which are components of TLR activated pathways leading to pro-inflammatory cytokine production. While neither DBT nor PCP have previously been shown to stimulate TNF-α production, they both increase production of two other critical pro-inflammatory cytokines, IL-1β and IL-6 (Martin et al. 2019a, b; Martin et al. 2019a, b; Sushak et al. 2020). TBT- and PCP-induced stimulation of IL-1β and IL-6 are at least partially dependent on TLRs (Alcala et al. 2022; Seaton-Terry et al. 2025). The current study shows that both DBT and PCP are also able to stimulate TNF-α production by immune cells. Continuous TNF-α elevation driven by TBT, DBT, or PCP exposure can contribute to immune dysregulation, tissue damage, and increased risk for autoimmune and malignant disease (Adegbola et al. 2018; Balkwill and Mantovani 2001; Barcia et al. 2011; Farrugia and Baron 2016; Fischer and Maier 2015; Frankola et al. 2011; Macarthur et al. 2004; Rauf et al. 2022). A recent study investigating increases in chronic inflammation seen with aging (inflammaging) indicates that this phenomenon may be primarily attributable to industrialization (including the increased exposure to man-made chemicals such as those in the current study) rather that to age (Franck et al. 2025).

As mentioned earlier, TBT stimulation of pro-inflammatory cytokine production involves activation of MAPKs (Brown et al. 2018; Lawrence et al. 2018). Because of its lipophilic properties (Arnold et al. 1997), TBT can enter the cell to directly activate intracellular components such as MAPKs. Previous studies have shown that TBT exposures activate MAPKs in immune cells (Aluoch and Whalen 2005). However, it could also interact in a direct or indirect fashion with TLRs and stimulate MAPKs through TLR signaling pathways. The current study shows that TBT stimulated TNF-α production relies, at least in part, on TLR2 and TLR4. It has previously been shown that TBT uses TLR4 and TLR8 as part of its mechanism of elevating the production of IL-1β and IL-6 (Alcala et al. 2022). Thus, the manner by which TBT leads to elevation of TNF-α seems to be slightly different than that seen with IL-1β and IL-6. However, elevation of all 3 cytokines involved TLR4. Like TBT, DBT has been shown to increase the production of IL-β and IL-6 from human immune cells in a MAPK dependent manner (Sushak et al. 2020). Although DBT’s hydrophobicity is lower than TBT’s (Ostrakhovitch 2022), it is still capable of entering the cell and directly activating MAPKs, it could also lead to MAPK activation by engaging one or more TLR resulting in downstream activation of MAPKs. Again, previous studies indicated that DBT exposures activate MAPKs in immune cell (Odman-Ghazi et al. 2010). The current study shows that DBT stimulation of TNF-α production is, in part, dependent on activation of both TLR1/2 and TLR4. The role of TLR4 in DBT elevation of TNF-α is like that seen with TBT. In contrast, DBT stimulation of TNF-α production was at least partially dependent on TLR1/2 while TBT used both TLR4 and TLR8. A previous study examining the role of TLRs in DBT-induced increases in IL-1β and IL-6 showed no consistent involvement of any of the TLRs in DBT’s stimulation of immune cell production of those cytokines (Seaton-Terry et al. 2025).

PCP is a hydrophobic organochlorine compound (Nowosielski and Fein 1998) used as a wood preservative, fungicide, and herbicide (Cirelli 1978). As mentioned earlier, levels of PCP as high as 5 µM are found in the serum of exposed individuals with levels of 0.15 µM found in those with no known exposures (Cline et al. 1989). The results of this study show that PCP-induced increases in TNF-α production in human immune cells involve TLR4. Inhibition of TLR4 consistently reduced cytokine secretion across donors, Additionally, the intracellular receptors, TLR3 and TLR8, also played a role in PCP stimulation of TNFα production. Studies examining the role of TLRs in PCP stimulation of the pro-inflammatory cytokines IL-1β and IL-6 showed that both TLR4 and TLR8 had a role in the PCP-induced stimulation of IL-6. However, PCP stimulation of IL-1β only relied upon TLR4 (Seaton-Terry et al. 2025). Thus, TLR4 has a role in the process by which PCP leads to elevation of each of these cytokines. However, PCP-induced increases in both IL-6 and TNF-α engage additional TLRs. This is likely due to the overlapping but distinct pathways by which each of these cytokines are regulated (Gaestel et al. 2009).

MyD88 is an adapter protein that directly interacts with activated TLRs (with the exception of TLR3) to translate receptor activation to downstream signaling components (Duan et al. 2022). The current study showed that inhibition of MyD88 function with TJ-M2010-5 consistently diminished the ability of TBT and PCP to elevate TNF-α production. DBT showed decreased capacity to stimulate TNF-α production in cells from 2 of 4 donors. The role of MyD88 in TNF-α stimulation by TBT and PCP is consistent with results from a previous study examining its role in IL-1β and IL-6 production, In that study, TBT, DBT, and PCP all showed a dependence on MyD88 in their stimulation of both IL-1β and IL-6 (Seaton-Terry et al. 2025). Thus, the reliance of compound-induced stimulation of IL-β, IL-6 and TNFα on MyD88 activation further substantiates the role that TLRs play in the elevation of pro-inflammatory cytokines seen with exposure to TBT, DBT, and PCP.

Each of the compounds examined likely have multiple cell surface and intracellular targets whose engagement leads to the observed alterations in pro-inflammatory cytokine levels. Individual variation in number and sensitivity of such targets may at least in part explain the variation seen among cells from distinct donors in terms of the concentrations of compound that stimulated TNF-α production as well as in the response to selective inhibition of TLRs. Each compound has the potential to activate cytokine production via certain TLR regulated pathways. This activation could occur by the compound entering the cell and directly activating MAPKs which are downstream components of TLRs pathways, including TLR4. Based on the current results it is possible that TBT, DBT, and PCP are stimulating the TLR pathway by direct interaction with the receptor as well as by entering the cell and more directly activating down-stream components of TLR pathways such as MAPKs. The fact that the adapter protein directly linked to TLR4 (as well as others), MyD88, caused very significant decreases in TBT- and PCP-induced TNF-α production, is a further indication of TBT and PCP having a more direct interaction with TLRs. Additionally, molecular docking analysis of PCP and DBT with the ectodomains of TLRs found that PCP and DBT are capable of binding to TLR4 (Seaton-Terry et al. 2025) .

In summary, the current study indicates that TBT, DBT, and PCP interact either directly or indirectly with TLRs to increase TNF-α production. TBT-, DBT-, and PCP-induced production of TNF-α is, at least partially, dependent on TLR4. Additionally, TBT-, DBT-, and PCP- induced stimulation of TNF-α was in part dependent on other TLRs: TBT-induced TNF-α production was somewhat dependent on TLR2, DBT-induced TNF-α production on TLR1/2 and PCP-induced TNF-α production on TLR 3 and TLR 8. Blocking the initial component of TLR1/2, TLR2, TLR4, and TLR8 signaling, MyD88, led to very significant blockade of TBT- and PCP-induced TNF-α production. These findings highlight the converging impact of environmental contaminants on immune regulation and emphasize the need for continued investigation into the molecular pathways linking environmental exposure to inflammatory disease.

## Data Availability

All data supporting the findings of this study are available within the paper or can be provided upon request.
